# Phylogeny and classification of the Catantopidae at the tribal level (Orthoptera, Acridoidea)

**DOI:** 10.3897/zookeys.148.2081

**Published:** 2011-11-21

**Authors:** Baoping Li, Zhiwei Liu, Zhe-Min Zheng

**Affiliations:** 1 Department of Entomology, Nanjing Agricultural University, Nanjing, Jiangsu, China; 2 Department of Biological Sciences, Eastern Illinois University, Charleston, IL61920, USA; 3 Department of Biology, ShaanxiNormal University, Xi’an, Shaanxi, China

**Keywords:** Orthoptera, Acridoidea, Catantopidae, China, Phylogeny, Morphology, Systematics, Male Genitalia

## Abstract

The grasshopper family Catantopidae is a well-known group, whose members include some of the most notorious agricultural pests. The existing classifications of the family are mostly utilitarian rather than being based on phylogenetic analysis and therefore unable to provide the stability desired for such an economically important group. In the present study, we present the first comprehensive phylogenetic analysis of the family based on morphology. By extensively sampling from the Chinese fauna, we included in the present analysis multiple representatives of each of the previously recognized tribes in the family. In total, we examined 94 genera represented by 240 species and evaluated 116 characters, including 84 for external morphology and 32 for male genitalia. The final matrix consists of 86 ingroup taxa and 88 characters. Our phylogenetic analyses resulted in a high resolution of the basal relationships of the family while showed considerable uncertainty about the relationships among some crown taxa. We further evaluated the usefulness of morphological characters in phylogeny reconstruction of the catantopids by examining character fit to the shortest trees found, and contrary to previous suggestions, our results suggest that genitalia characters are not as informative as external morphology in inferring higher-level relationship. We further suggest that earlier classification systems of grasshoppers in general and Catantopidae in particular most probably consist of many groups that are not natural due the heavy reliance on genitalia features and need to be revised in the light of future phylogenetic studies. Finally, we outlined a tentative classification scheme based on the results of our phylogenetic analysis.

Zhiwei Liu would like to dedicate this paper to the honor of Professor Kumar Krishna for his friendship, kindness, professional encouragement, and the good times at the AMNH.

## Introduction

Catantopidae (Acridoidea, Orthoptera) is a well-known grasshopper family; its members include some of the most notorious pests in agriculture, including *Schistocerca gregaria* (Forsköl), *Oxya* spp, and *Melanoplus* spp (Hill 1987). The family is by far the largest and the most diverse acridoid family, consisting of over 3000 species in about 640 genera mainly distributed in the tropical and subtropical areas of the world (Vickery and Kevan 1983).

The previous classifications of Acridoidea (Orthoptera) have been predominantly utilitarian; existing classifications of the superfamily almost entirely ignored phylogenetic relationships among taxa. Among the various classification systems or schemes of acridoids (Dirsh 1961, 1975, Harz 1975, Otte 1981, Yin 1982, Xia 1994, Li and Xia 2002) and several other classifications specifically proposed for the Catantopidae (Tinkham 1940, Mistshenko 1952, Harz 1975), there exist a great deal of disagreement concerning the classification within the family (Table 1), which cannot be easily settled because of the lack of phylogenetic studies. The most influential classification systems of Acridoidea at the present are still the one established by Dirsh (1956) and its modified versions (Dirsh 1961, 1975). The classifications by Dirsh are based on extensive comparative studies of the genitalia morphology of both sexes as well as other morphological characters, emphasizing especially the importance of the morphology of phallic complex and epiphallus in defining higher taxa. Several other authors also proposed their own classification for the Acridoidea (Rehn and Grant 1960, Uvarov 1966, Jago 1971, Vickery and Kevan 1983, Liu^ 1991^). Otte (1981, 1984) adopted a compromised version of the various systems in his monographic treatment of North American grasshoppers. These classifications, although different, have one thing in common: all are entirely based on overall similarity and make little, if any, reference to phylogenetic relationship.

**Table 1.** Classification systems of the Catantopid fauna from China

Tinkham (1940)	Mistshenko (1952)	Dirsh (1961), Uvarov (1966)	Harz (1975)	Dirsh (1975)	Yin (1982)	Xia (1994)	Otte (1981), Eades et al. (2011)^1^
Cyrtacanthacrinae	Catantopinae	Acrididae	Catantopidae	Hemicarididae	Oedipodidae	Catantopidae	Acrididae
				Hemiacridinae			
	Conophymatini	Hemiacaridinae		Conophyminae	Conophyminae	Conophyminae	Conophyminae
Spathosternini			Spathosterninae	Spathosterninae	Spathosterninae		Spathosterninae
Leptacri				Leptacrinae			Leptacrinae
Caryandae				Catantopidae			
	Dericorythini	Dericorythinae	Dericorythinae	Dericorythinae	Dericorythinae	Dericorythinae	(Dericorythidae)
Oxyae	Oxyini	Oxyinae	Oxyinae	Oxyinae	Oxyinae	Oxyinae	Oxyinae
Catantopini	Catantopini	Catantopinae	Catantopinae	Catantopinae	Catantopinae	Catantopinae	Catantopinae
Calliptamini	Calliptamini	Calliptaminae	Calliptaminae	Calliptaminae		Calliptaminae	Calliptaminae
Eyprepocnemini	Eyprepocnemidini	Eyprepocneminae	Eyprepocneminae	Eyprepocneminae		Eyprepocneminae	Eyprepocnemidinae
Cyrtacanthacridini	Cyrtacanthacridini	Cyrtacanthacridinae	Cyrtacanthacridinae	Cyrtacanthacridinae		Cyrtacanthacridinae	Cyrtacanthacridinae
Coptacrae	Coptacrini	Coptacrinae		Coptacrinae		Coptacrinae	Coptacridinae
Podisminae	Podismini			Podisminae	Podisminae	Podisminae	Podismini
	Tropidopolini	Tropidopolinae	Tropidopolinae	Tropidopolinae			Tropidopolinae
Tristrini	Tristriini					Tristrinae	(Tristiridae)
	Hieroglyphini					Hieroglyphinae	Hieroglyphinae
Trauliae	Trauliini						
Oxyrrhepini					Habrocneminae	Habrocneminae	Habrocneminae
Xenacanthippi						Melanoplinae	Melanoplodinae
Tauchirae						Acrididae	Acridinae
Incolacri						Leptacrinae	
	Egnatiinae	Egnatiidae	Egnatiinae	Egnatiinae	Egnatiinae	Egnatiinae	Egnatinae
			Acrididae		Gomphoceridae	Gomphoceridae	Gomphocerinae

1. Additional subfamiliies of Acrididae: Cpiocerinae, Eremogryllinae, Euryphyminae, Leptysminae, Marellinae, Oedipodinae, Ommatolampinae, Pauliniinae, Protolabinae, Rhytidochrotinae, Teratodinae

The need for a classification of the grasshoppers and locusts based on phylogeny, rather than based on overall similarity, is obvious. Yin (1982) pointed out the importance of distinguishing between plesiomorphic and derived features in the classification of the acridoids and paid special attention to the transformation series of antennae, wings, and stridulating apparatuses and tympanum. However, Yin’s classification of Acridoidea based on his studies of the Chinese members of the group was not based on phylogenetic analysis and his circumscriptions of higher-rank taxa were often based on characters that obviously have been obtained through convergent evolution. Key and Colless (1993) attempted to conduct a cladistic (and phenetic) analysis of the subfamily Catantopinae from Australia. They coded 104 male external characters for 166 genera and conducted a series of analyses from typical phenetic approaches to cladistic methods as implemented in PAUP (version not mentioned).The results of this particular study showed almost no resolution of relationships or useful clustering except for a few ‘low-level groups’. The authors consequently did not even bother to present the cladograms and resolved to ‘develop a classification by traditional non-quantitative methods’.

There has been an increased interest in recent years in the phylogenetic relationship of orthopteroid insects in general (Flook and Rowell 1997a, 1997b, 1998, 1999; Flook et al. 2000, Rowell and Flook 1998, Yin et al. 2003) and the acridoids in particular (Amedegnato et al. 2003, Chapco et al. 2001, Litzenberger and Chapco 2001, 2003, Ren et al. 2002, Xi and Zheng 1997, Xu et al. 2003, Xu and Zheng 1999, 2002; Zheng and Qiao 1998). Most of these recent studies are based on molecular data with relatively limited taxon sampling; the few morphology-based studies either targeted at lower level relationship (e.g., within genus, Xu et al. 2003, but see Song and Wenzel 2007) or are characterized by sporadic taxon sampling (Ren et al. 2002, Xu and Zheng 1999, 2002; Zheng and Qiao 1998). Therefore, the potential of morphology in resolving higher-level phylogeny within Orthoptera and Acridoidea has not been fully explored.

This lack of higher-level phylogenetic study of Catantopidae leads to a lack of stability in the classification within the family (Table 1), which is unusual for such a well-known and economically important group. In this paper, we present the first comprehensive phylogenetic analysis of the family Catantopidae based on morphology by sampling extensively from the Chinese fauna. Our purpose is to (1) conduct an exploratory phylogenetic analysis of the phylogenetic relationship within the family represented by the Chinese members, (2) provide an objective evaluation of the usefulness of morphological characters in phylogeny reconstruction of the acridoids in general and the catantopids in particular, and (3) provide a general framework for taxon sampling in future studies of acridoid phylogeny on a global basis.

## Materials and methods

### I. Monophyly

The name Catantopidae, or its original form Catantopinae as subfamily, has had a long history of divergent usages (Key and Colles 1993). The modern definition of Catantopidae took after the name of Cyrtacanthacrinae (Tinkham 1940, Roberts 1941) and was subsequently assigned subfamily status as Catantopinae by Mistshenko (1952). The latter author further assigned the members of the subfamily in the fauna of the former Soviet Union and adjacent countries into thirteen tribes, and considered Egnatiinae as a separate group from the Catantopinae. Mistshenko (1952) was mostly accepted by later authors, including Dirsh (1961), Uvarov (1966), and Harz (1975). Dirsh (1975) later divided Mistshenko’s Catantopinae into two families, Hemiacrididae and Catantopidae, and included Egnatiinae in the family Catantopidae. Yin (1982) also divided Mistshenko’s Catantopinae into two families, Acrididae and Oedipodidae, but treated Egnatiidae as a separate family. Xia (1994) included in the family Catantopidae some of the subfamilies of Oedipodidae in Yin’s system and raised most of the tribes in Mistshenko’s system to subfamilies. The Xia System has been adopted for the recent monographic treatment of the Chinese fauna of Catantopidae (Li and Xia 2002). The classification scheme used by Otte (1981, 1984) in his monographic treatments of the grasshoppers of North America north of the Gulf of Panama, although unexplained, is obviously completely utilitarian without reference to phylogenetic relationship among groups. The Otte classification was later expanded and adopted by the author in his multi-volume catalog of the orthopteran insects of the world (Otte 1994a, 1994b, 1994c, 1995a, 1995b), which in turn has been eventually published as a searchable online database, the Orthoptera Species File (Eades et al 2011). While the Orthoptera Species File database is tremendously useful for taxonomic purposes, species groups defined by earlier classification systems were often used in phylogenetic studies on Acrididae / Acridoidea at levels of tribe and above (Litzenberger and Chapco 2003; Song and Wenzel 2007). A comparison of catantopid classification systems by various authors is given in Table 1.

Catantopidae in our view is readily defined by the unmistakable synapomorphy of having a distinct prosternal process between the forecoxae. Although some species of Pamphagidae and Pyrgomorphidae have a lamellate process on the prosternum, the process in these species is on the anterior margin of the prosternum and is obviously an independently evolved feature not homologous to the prosternal process between the forecoxae observed in Catantopidae. Nonetheless, as shown in Table 1, there was considerable disagreement among earlier authors about the definition of Catantopidae, which obviously arose from the fact that earlier acridologists defined higher-level taxa on basis of overall similarities, instead of on synapomorphies. Our interpretation of Catantopidae in the present paper, as defined by the presence of prosternal process between the forecoxae, is in accordance with Catantopinae of Roberts (1941) and Mistshenko (1952) and Catantopidae of Harz (1975) and is equivalent to the “Spine-breasted Acrididae” as keyed out in Otte (1981). Throughout the paper, we consistently use the name Catantopidae except when discussing its treatment by various previous authors. In the latter case, they were referred to as were originally treated by these authors, such as Catantopinae or Catantopini. The same rule is also consistently applied to other taxa, e.g. Egnatiidae.

### II. Taxa Sampling and Sources of Specimens

About 327 species in 96 genera of Catantopidae (*sensu* Mistshenko 1952) are known from China, with representatives from both the Palearctic (21.44%) and the Oriental regions (79.56%) (adjusted according to Huang and Chen (1999)). The Chinese fauna of catantopids represents 15% of world genera of the family (data from Vickery and Kevan, 1983) and all of the tribes recognized by Mistshenko (1952) or subfamilies by Dirsh (1975). In this study, we examined a total of 2,536 specimens representing 240 species in 94 genera, accounting for 73% and 98% of the total number of species and genera known from the country, respectively. Of the 94 examined genera, 84 genera were included in our phylogenetic analysis while the other eight were excluded (Appendix 1). The reasons for the exclusion are: 1) type specimens were not available for examination and no other specimen of these genera had been collected since the original publications, such as *Tzacris* Tinkham and *Chapacris* Tinkham; 2) only females were then discovered, such as *Liaopodisma* Zheng. In addition, we also left out several genera that were described after the data collection stage of this study, such as *Caryandoides* (Liu and Li 1995, Özdikmen 2009) and *Tectiacris* (Wei and Zheng 2005). The final inclusion of taxa represented all of the tribes recognized by Mistshenko (1952) and subfamilies by Dirsh (1975).

The majority of the study materials of the present project were provided by the following institutions (curators in parentheses):

Entomological Museum, Shaanxi Normal University, Xi’an, Shaanxi Province (Shengquan Xu)

Entomological Museum, Zhongshan University, Guangzhou, Guangdong (Geqiao Liang)

Entomological Museum, Research Institute of Entomology, Chinese Academy of Sciences, Shanghai (Kailing Xia)

Entomological Museum, Beijing Institute of Zoology, Chinese Academy of Sciences, Beijing (Chunmei Huang)

Zoological Museum, Northwest Plateau Institute of Biology, Chinese Academy of Sciences, Xining, Qinghai (Xiangchu Yin)

### III. Selection of outgroups

Because of the lack of phylogenetic analysis of Acridoidea at levels above subfamily, we had to rely on previous systematic studies on Acridoidea for outgroup selection. All existing classifications of Acridoidea treated Catantopinae, Egnatiinae, Acridinae, and Oedipodinae as being closer to each other than they are to Pyrmorphinae and Pamphaginae (Roberts 1941, Mistshenko 1952, Dirsh 1956, 1961, 1975; Yin 1982, Xia 1994). Dirsh (1961, 1975) suggested that Egnatiinae was closer to Catantopinae than any other subfamily of his Catantopidae because Egnatiinae possesses a Comstock-Kelogg gland, which is otherwise believed to occur only in Catantopinae. Furthermore, Egnatiinae and Catantopinae share similar folds and sculpture in the internal surface of foregut, which are different from those of Oedipodinae. Stebaev et al. (1984) also agreed on a close relationship between Egnatiinae and Catantopinae on basis of cytogenetical, taxonomical and ecological data, but considered the Egnatiinae as a tribe within Catantopinae. Many contemporary acridologists are in agreement about a close relationship between Egnatiinae and Catantopinae (e.g., David Hollis, pers. comm.). Because of the close relationship between Egnatiidae and Catantopidae, very likely as sister clades, and the lack in Egnatiidae of the obvious catantopid synapomorphy of having a prosternal process between the forecoxae, the family Egnatiidae represented by the two genera *Egnatius* Stal and *Egnatioides* Voss, was used as outgroup for the phylogenetic analysis of Catantopidae relationships.

### IV. Specimen study and character coding

Terms and abbreviations used in the present study followed B.-Bienko and Mistshenko (1951) for external morphology and Dirsh (1956, 1961, 1975) for genitalia structures.

Specimens for the study were selected in the following order of priority: 1) type specimens, 2) specimens determined by the author of the taxon, and 3) specimens determined by experts of the taxon. All characters were coded from direct observation of specimens, except in a few instances where characters of a species were coded based on illustrations and descriptions from monographs or reviews (Willemse 1956, 1957; B-Bienko and Mistshenko 1951, Mistshenko 1952, Hollis 1975).

External morphology was surveyed before specimens were dissected for examination of genitalia characters. When available, multiple individuals were examined for each species and multiple species for each genus. For polymorphism at species level, we took an approach similar to, but much more restricted than, the “majority state rule” proposed by Wiens (1995). We generally avoided characters that are polymorphic at species level, and only in very few cases, coded species in question as the predominant state when the other state(s) was rare (presence rate < 15%). In a few cases when character polymorphism occurred at generic level, the characters in question were initially coded as missing for the genus, but were eventually abandoned and not included in the analysis. Some of the characters with three or more states were treated prior to the cladistic analysis as ordered or additive characters, *i.e.,* the transformation series was hypothesized to be 0-1-2 and so on. This was done only when it was possible to order the states unambiguously, e.g., for measurement ratios, and ordered characters are indicated in Appendix 1. In a few cases, one of the states of a main character was more finely subdivided into one or two subsidiary character(s). Taxa with other states of the main character were coded as having state unknown (character not applicable) in the subsidiary character. This commonly used method has been referred to as ‘state-unknown coding’ (Nordlander et al. 1996). The method may give incorrect lengths for some trees when there is homoplasy in the main character and different subsidiary states are ancestral for the different clades having the subdivided state of the main character (Maddison 1993). It has been suggested to use step matrices to represent main/subsidiary character systems exactly (Maddison and Maddison 1992), but this will slow down calculations considerably and is especially impractical for relatively large matrixes like ours. In the present study, therefore, we consistently used state-unknown coding for main/subsidiary character systems and weighted all main and subsidiary characters equally. More detailed discussion about the application of the method is found in Nordlander et al. (1996).

The final matrix contained 87 terminals, including outgroup and 86 catantopid genera in the ingroup, and 88 characters, of which 79 were phylogeny-informative and the other nine were autapomorphies (Appendix 2-3). The autapomorphic characters were excluded from the final cladistic analyses and not counted when calculating tree length, CI, or RI. Nonetheless, they were kept in the matrix for their taxonomic values and potential use in future phylogenetic studies involving the included taxa.

### V. Phylogenetic Analysis

PAUP version 4.0 beta10 (Swofford 2003) was used for phylogenetic analyses. The large number of taxa and characters included in this study did not allow the use of exact searching algorithms. Therefore, we used a combination of several ‘shortcut’ approaches. We first used PAUPRat (Sikes and Lewis 2001) to generate batch files for maximum parsimony analysis within PAUP using the Parsimony Ratchet method described by Nixon (1999). We performed 30 repetitions of the Parsimony Ratchet analysis, with 200 iterations per run as suggested by Sikes and Lewis (2001), giving a total of 6,000 iterations. The single shortest tree from each of the 6,000 iterations were then loaded into computer memory for comparison and only the shortest trees over all iterations were kept and duplicates of trees were removed. Because these overall shortest trees were each only the single best tree retained from a particular iteration, they each were probably one of the many possible equally most parsimonious trees or one of the less than most parsimonious trees that actually exist for the dataset. Therefore, these trees were further subjected to TBR branch swapping in order to find out all possible trees of equal or shorter length. To ensure that we find the best trees, we also analyzed our dataset in NONA 2.0 (Goloboff 1999a), a program said to be much faster than competitors like PAUP (Goloboff 1999b). For NONA analyses, we started with MULT*50 (randomize order of taxa, create a weighted Wagner tree, swap using TBR, and with 50 replications) and then swapped the shortest trees from MULT analysis using Max*, which is equal to PAUP’s TBR swapping. NONA was also used for calculation of Bremer Support values (/decay index) for branches (Bremer 1994) while PAUP was used for diagnosis of apomorphic characters supporting each branch. TNT (Goloboff et al. 2011), a program that implemented the tree search methods of NONA as well as additional search methods, including sectorial search, tree drifting, and tree fusing (Goloboff 1999b), was also used for Parsimony Ratchet analysis of the dataset with options comparable to afore-described NONA analysis. The other so-called “New Technology” searching techniques were also used with default options of the software, but were not extensively explored because our dataset was not too large and thus further aggressive approximation was not considered necessary.

## Results

### I. Character Analysis

We examined a total of 116 characters, including 84 characters of external morphology and 32 characters of male genitalia morphology. Twenty-eight characters were excluded from out analysis either because they were too variable across examined species of a genus to reach a generic consensus or because they were continuous and discrete coding of character states was impossible. In addition, characters of body color patterns, although important for identification of some species of the family, were found to be too variable, both among individuals of species and among species of genera, to be of much use in resolving phylogenetic relationships within Catantopidae and were therefore excluded from the present study. The eighty-eight characters included in the final character matrix consist of 71 external morphological characters and 17 genitalia characters (Appendix 2). Character fit on the shortest trees, as expressed by the consistency index (CI) and retention index (RI), was lower for characters of male genitalia morphology in comparison to characters of external morphology (Table 2).

**Table 2.** Fit on shortest trees of different categories of characters, as expressed by the consistency index (CI) and retention index (RI) (n = number of characters; autapomorphis excluded).

Character Category	n	CI	RI
External Morphology	63	0.19	0.58
Body shape	1	0.25	0.63
Head	10	0.17	0.54
Mesosoma	29	0.20	0.66
Metasoma	23	0.20	0.45
Male Genitalia	16	0.12	0.49

### II. Phylogenetic Analyses

**Figure 1.**
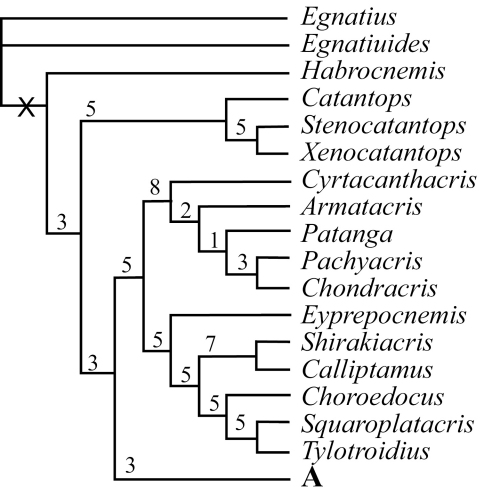
Strict consensus tree of the 22,355 found shortest trees using Parsimony Ratchet method in PAUP 4.0 beta10 (30 repetitions and 200 iterations per run, followed by TBR swapping). Above each resolved branch is the Bremer Support value (/decay index) for the branch estimated using NONA2.0. Only the completely resolved basal part is shown.

**Figure 2.**
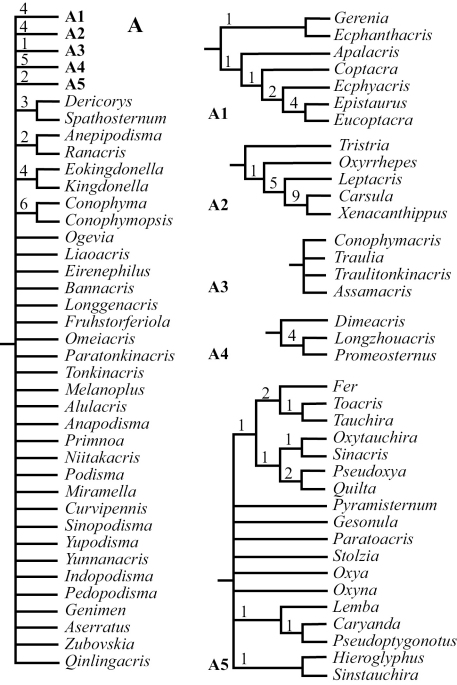
Strict consensus tree of the 22,355 found shortest trees using Parsimony Ratchet method in PAUP 4.0 beta10 (30 repetitions and 200 iterations per run, followed by TBR swapping). Shown in the figure is the expansion of Clade A of Figure 1. Several completely resolved clades are further expanded as **A1, A2, A3, A4** and **A5** respectively.

**Figure 3.**
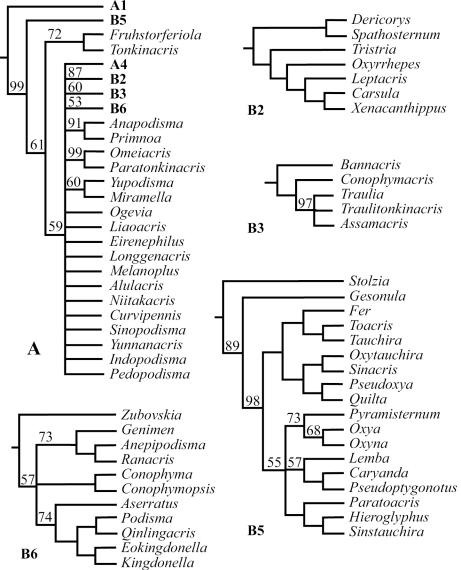
Majority (50% and above) consensus tree of the 22,355 found shortest trees using Parsimony Ratchet method in PAUP 4.0 beta10. The basal part of the majority consensus tree is completely resolved and is the same as in Figure 1, and the figure shows only the phylogenetic relationship within Clade **A** as resolved by MJ consensus tree. The clades better resolved in comparison with strict consensus tree are further expanded as B**2**, **B3**, and **B5**. **B5** is the same as **A5** of Figure 2, but with better internal resolution. **B2** is **A2** plus (Dericorys, Spathosternum) at the base, and **B3** is **A3** plus Bannacris added at base and has higher internal resolution. **B6** consists of several pairs of genera unresolved in the strict consensus tree. **A1** and **A4** are each completely resolved and remain the same as in Figure 1, and are thus not expanded here in. More differences between strict and MJ consensus trees are found in the basal part of Clade **A** (cf. Figure 2: **A**). Number above each branch is frequency of occurrence of a particular branch among all 22,354 found shortest trees, and branches not indicated with a number have 100% occurrence.

Using maximum parsimony analysis with Nixon’s ratchet method, we found in thirteen of our 30 replications and 218 of the 6,000 iterations a tree with the shortest length of 688 steps (L=688, CI = 0.17, RI = 0.55). With duplicate trees deleted, the final number of the shortest trees was 204;subsequent swapping of these optimal trees using TBR did not find shorter trees, but found a total of 22,354 equally most parsimonious trees. Figs. 1–2 and Fig. 3 show the strict consensus tree with Bremer Support for completely resolved branches and the 50% majority consensus tree with percentage of branches appearing in all shortest trees summarized, respectively.

Searching with NONA 2.0 (Hold=10,000–30,000, Mult*50, and Max*) did not find trees shorter than those found with PAUP 4.0 using parsimony ratchet method. Although we were always able to find trees of the shortest length in a few minutes with NONA, our searches invariably resulted in only about 50 trees with MAX*, even when we increased the number of trees to be held in memory to 30,000. Further swapping using SSWAP*2 and MSWAP*2 apparently would take a long time (3.2GHZ CPU frequency and 1G RAM) and were terminated after a few hours. Comparison of the NONA trees with PAUP trees showed that they were a (small) subset of the trees we found using ratchet method in PAUP. Searching with TNT, either ratchet method or other new technology methods, did not resulted in shorter trees.

### III. Phylogenetic Relationship

Although the number of shortest trees found by our cladistic analyses is huge, the phylogenetic relationship among genera at the base of the cladogram was well resolved, and all basal clades were also relatively well supported with Bremer Support values ranging mostly from 3 to 8 (Fig. 1). The majority of genera, 71 out of 88, fell into the monophyletic Clade A (Fig. 1), which is a polytomy consisting of several relatively well-supported monophyletic clades (Fig. 2: A, A1–5; clade A3 is only supported by a Bremer Support value of 1) as well as a number of unresolved genera / pairs of genera (Fig. 2: A). When a 50% majority consensus tree was calculated, better resolution within Clade A is achieved (Fig. 3, A, B2–B6). In comparison to the strict consensus tree, a sister relationship between A1 and the rest of the clade is supported by 99% of all shortest trees (Fig. 3: A), and A5 (Fig. 2: A5) is supported as the sister clade of the clade consisting of the rest of the genera with improved within-clade resolution (Fig. 3: B5), and (*Fruhstorferiola* + *Tonkinacris*) becomes the sister clade to the clade including all members of Clade A except clade A1 and B5 (Fig. 3: A). This terminal clade, while supported by 59% of all shortest trees, form a polytomy consisting of several monophyletic, relatively well resolved clades, 12 distinct genera, and three genera pairs. In addition, there is also an increased resolution at the base of Clade A -- B2 consists of A2 and (*Dericorys* + *Spathosternum*) (Fig. 3: B2), B3 includes A3 and *Bannacris*, and an additional clade is resolved (Fig. 3: B6).

### IV. Discussion

Male genital morphology received special attention from Dirsh (1956, 1961, and 1975) when the author established his classification of acridoids. In fact, the various versions of Dirsh classification depended heavily on the male genitalia morphology, and the practice has greatly influenced later systematists of grasshoppers and other orthopteran insects (Hollis 1975, Yin 1982, Ronderos and Cigliano 1991, Xia 1994, Zheng and Xia 1998). Our result showed that character fit on the shortest trees, as expressed by the consistency index (CI) and retention index (RI), was actually lower for characters of male genitalia morphology in comparison to external morphology characters (Table 2), suggesting that genital characters are not as phylogeny informative as previously thought, at least at higher level, and earlier classification systems of grasshoppers in general and Catantopidae in particular probably include many groups that are not natural due to the heavy reliance on genital features. Eberhard (1985) argued that the species-specific diagnostibility of male genitalia is a reflection of both the rate and extent to which they diverge, and any structure so useful taxonomically must have evolved rapidly. In fact, a recent study showed that morphologically very similar species of *Melanoplus* grasshoppers differ in the shape of the male genitalia and this is probably due to extremely rapid speciation caused by glacial cycles during the Pleistocene glaciations (Knowles and Otte 2000). The rapid evolution of male genitalia morphology is considered to be caused by strong sexual selection on the male imposed by the females (Eberhard 19985, Knowles and Otte 2000). Regardless of the mechanism, male genital features, while very useful in species identification, show high degree of homoplasy and are therefore of limited value in phylogenetic studies, especially at higher levels. Consequently earlier classifications of acridoids as well as Catantopidae need to be revised critically in the light of phylogenetic analyses based on a broad range of characters.

An earlier attempt to study the phylogenetic relation within Catantopidae from Australia found almost no resolution, especially at the base (Key and Colless 1993), which is strikingly different from the results of our study where the phylogenetic relationship was reasonably resolved, especially at the base. Key and Colless (1993) was able to assemble an impressive dataset consisted of 166 terminals and 104 characters, but unfortunately provided otherwise very limited information about their dataset, which prevents us from interpreting exactly why there is such a big difference between their results and ours. Several factors might have contributed to this. For example, their study is based on males only. While male characteristics are frequently the only useful features for species identification, especially for closely related species, males of different grasshopper species may have been subjected to sexual selection and developed convergent similarities similar to what we have discussed earlier about male genital characteristics. In addition, the authors only used Neighbor-joining and Wagner parsimony without further branch swappingin their analyses, and it is thus very unlikely that what the authors found were the shortest trees. It would be of interest to request from the authors their dataset and reanalyze it using the currently available computation power that is far more superior than it was almost two decades ago. Computation power is especially relevant for analyzing dataset of their size.

**Figure 4.**
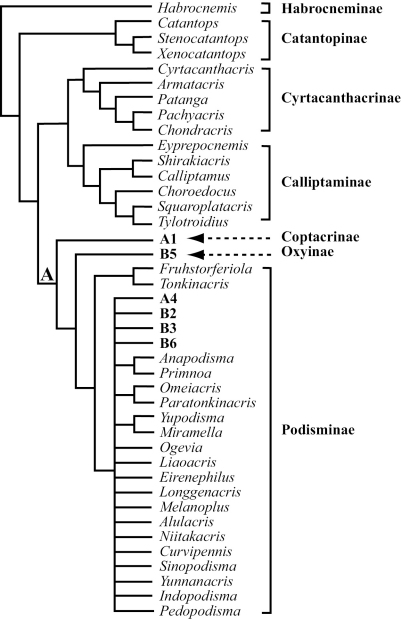
A possible scheme classification of Catantopidae from China based on parsimony phylogenetic analysis of 86 genera and 79 phylogeny–informative morphological characters. Details of Coptacridae and Oxynae are found in Figure 2 (Clade **A1** Coptacridae and **A5** Oxynae) and Figure 3 (**B5** Oxynae). Podisminae is further divided into six tribes, of which five are supported as monophyletic by the 50% majority consensus tree of all shortest trees found while the other ‘tribe’ Melanoplini is suggested as a ‘sink’ to temporarily keep the genera that do not belong to any of the supported clades. The Fruhstorferiolini is the most basal tribe consisting of Fruhstorferiola and Tonkinacris, while details of Melanoplini are found in Figure 2 (**A4**: Promeosternini) and Figure 3 (**B2** Dericorythini, **B3** Traulini, **B6** Podismini).

Rowell and Flook (1998) presented a phylogenetic tree for the Acridoidea based on the mitochondrial DNA sequences 12S and 16S. They found support for several catantopid clades, *i.e.*, Oxyinae, Podisminae+Melanoplinae, and Coptacridinae. In addition, their study also supported as monophyletic the clade consisting of Cyrtacanthacridinae, Calliptaminae, Catantopinae *s. str.*, and Eyprepocnemidinae. These clades are mostly supported by the present study except the monophyly of (Cyrtacanthacridinae + Calliptaminae + Catantopinae *s.s*. + Eyprepocnemidinae). While a sister relationship between Cyrtacanthacridinae and Calliptaminae is supported by the present study, Catantopinae is supported as a monophyletic basal clade in the family cladogram and *Eyprepocnemis* as a member of Calliptaminae (Fig. 4).

Rowell and Flook (1998) also suggested that the Acridoidea ‘seems to be the product of a single explosive radiation’ because they were unable to find a resolution at the subfamily level for the basal acridoids. However, this conclusion, according to the authors, is based on a ‘preliminary analysis’, for which the method was not described, and therefore has to be treated with caution. Meanwhile, the result of the study may be biased simply because of the used genes being inadequate with regard to the divergence level and evolution rate of the study group. According to Simon et al. (1994), the mitochondrial rRNA genes of 12S and 16S are considered to be mostly useful at the population level where highly variable sites have not yet experienced multiple substitutions and at deep levels of divergence where the more conserved sites of these genes supply useful phylogenetic information. At intermediate levels of divergence, however, the relatively variable sites probably have experienced multiple substitutions that may obscure phylogenetic signals. In addition, the rates and patterns of evolution of mitochondrial rRNA genes can vary greatly among taxa (Simon et al. 1994, and references therein). The particular analysis of Rowell and Flook (1998) of Acridoidea based on these two genes might just deal with this ‘intermediate level of divergence’ for the Orthoptera. It would be interesting to reanalyze their dataset to resolve the phylogenetic relationship at various levels with in the superfamily, e.g., to include all their major lineages, but include only a few of their sampled species for each of these lineages, or alternatively, analyze each of these major lineages with all their sampled species included. Unfortunately, the article provided neither the sequences nor genbank accession numbers for the sequences.

To our knowledge, the present study is the most comprehensive of its kind to study the higher level phylogeny of orthopteran insects in terms of the number of taxa sampled and characters examined and coded. Through this study we were able to demonstrate that the external morphology of orthopteran insects can be a very useful source for assessing higher-level phylogeny. For example, the study provided complete resolution for the basal relationships of the Catantopidae (Fig. 1), Nonetheless, our dataset were unable to provide an unambiguous solution for the relationships within the largest terminal clade that comprise 80% of all sampled genera in this study (Figs. 2, 3).It is generally accepted that phylogenetic hypotheses basing on as many independent lines of evidence as possible have the highest explanation value (Nixon and Carpenter 1996a). Combining morphological and molecular data should be the direction for future phylogenetic studies of orthopteran insects including Catantopidae. In addition, our study sampled only taxa from China, which was necessary due to the lack of resources, and future phylogenetic studies of Catantopidae should include representative taxa from other areas of the world.

### V. Classification of Chinese Catantopidae

Based on the strict consensus tree and the 50% Majority-rule consensus of the 22,355 shortest trees, we hereby outline a scheme for the classification for the family Catantopidae from China. As we discussed above, a comprehensive phylogenetic study based on a more inclusive taxon sampling from all regions of the world and including both morphology and molecular sequences is needed for highly resolving the phylogenetic relationship within the family, especially with regard to the relationship between and within the subfamilies Coptacridinae, Oxyinae, and especially Podisminae (see below). Therefore, the purpose of our outline is to serve as a basis for further studies, rather than as formal classification.

According to this scheme, the Chinese Catantopidae can be classified into seven subfamilies: Habrocneminae, Catantopinae, Cyrtacanthacrinae, Calliptaminae, Coptacridinae, Oxyinae, and Podisminae (Fig. 4). Among the seven recongnized subfamilies, Habrocneminae, Catantopinae, Cyrtacanthacrinae, and Calliptaminae are unambiguously supported as monophyletic clades, and the relationship of each to the rest of the family are completely resolved (Fig. 1, Fig. 4). Coptacridinae and Oxyinae, although each relatively well supported as monophyletic clade, are part of a crown clade that is highly unresolved in terms of within clade relationship (Clade A, Fig. 2). The monophyly of Podisminae, and the resolution of its relationship with Coptacridinae and Oxyinae are only supported by the 50% Majority-rule consensus, which is considered as a compromised solution in phylogenetic systematics (Nixon and Carpenter 1996b). Our analyses also identified within the subfamily Podisminae five monophyletic clades (Fig. 4), which may be treated as tribes: Fruhstorferiolini, Promeosternini, Dericorythini, Traulini, and Podismini. Finally, the rest of the genera within Podisminae are temporarily lumped together in the tribe ‘Melanoplini’ for convenience until further phylogenetic information becomes available.

## Figures and Tables

**Figures 5–9.**
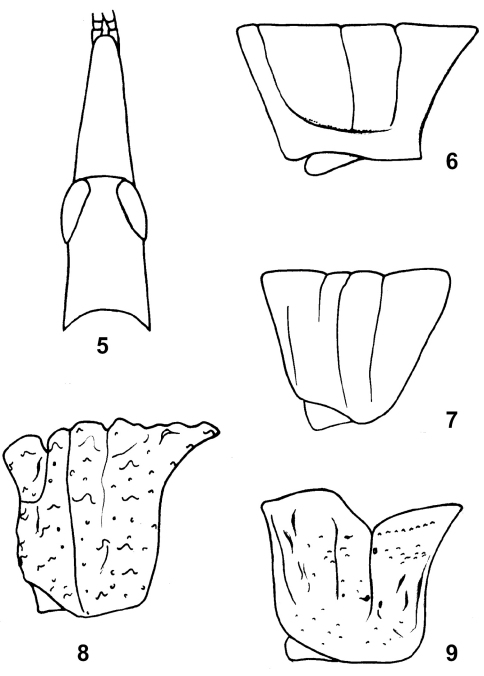
Head and pronotum: **5–6** *Carsula brachyptera* Huang et Xia, female: head and pronotum, dorsal view and lateral view, respectively; **7** *Yunnanacris yunaeus* (Ramme), pronotum, lateral view; **8** *Ecphanthacris mirabiis* Tinkham, male, pronotum, lateral view; **9** *Dericorys roseipennis* (Redt.), male, pronotum, lateral view.

**Figures 10–14.**
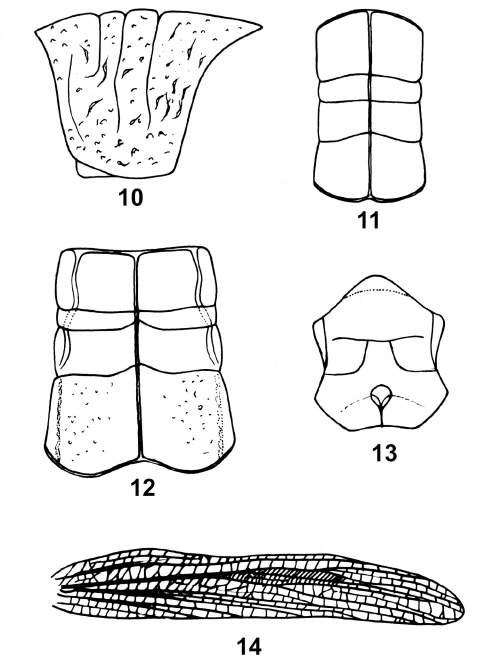
Thorax: **10** *Chondracris rosae rosae* (De Geer), male, pronotum, lateral view; **11** *Caryanda elegans* **I** –Bol., male, pronotum, dorsal view; **12** *Niitakacris rosaceanum* (Shiraki), male, pronotum, dorsal view; **13** *Longzhouacris hainanensis* Zheng et Liang, male, mesosternum and metasternum, ventral view; **14** *Tauchira damingshana* Zheng, female, elytron.

**Figures 15–23.**
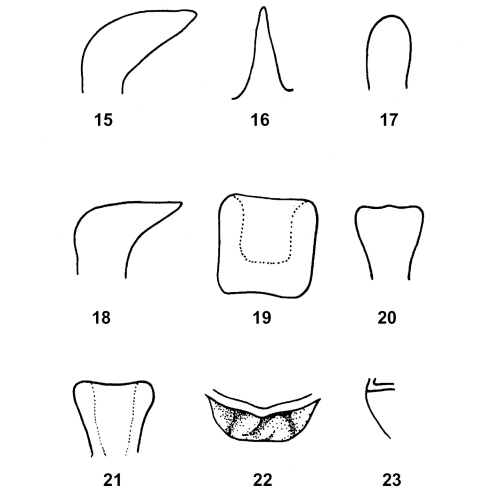
Prosternal process: **15** *Cyrtacanthacris tatarica* L., male lateral view; **16** *Caryanda elegans* **I** –Bol., male lateral view; **17** *Calliptamus barbarus* (Costa), male lateral view; **18–19** Tristria pulvinata Uv., male lateral view and ventral view respectively; **20** *Sinstauchira yunnana* Zheng, male rear view; **21** *Spathosternum prasiniferum* (Walk), male front view; **22–23** *Conophymopsis labrispinus* Huang, male ventral view, and lateral view respectively (from Huang 1983).

**Figures 24–28.**
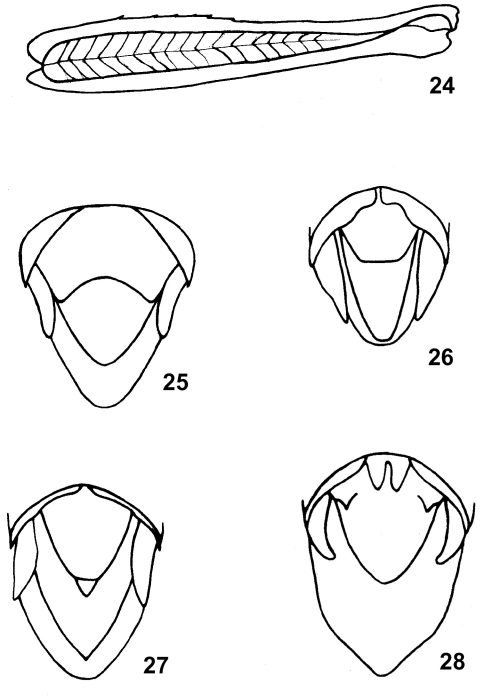
**24** *Dericorys roseipennis* (Redt.), male, hind femur, lateral view; **25–28** End of male abdomen in dorsal view: **25** *Dericorys roseipennis* (Redt.); **26**.*Ecphanthacris mirabiis* Tinkham; **27** *Spathosternum prasiniferum* (Walk); **28** *Anapodisma miramae* Dov.–Zap.

**Figures 29–35.**
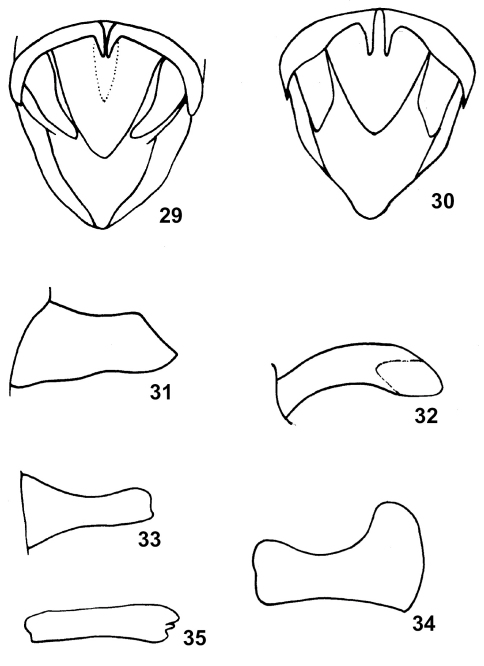
**29–30** End of male abdomen in dorsal view: **29** *Niitakacris rosaceanum* (Shiraki); **30** *Indopodisma kingdoni* (Uv.); **31–34** Cercus in lateral view: **31** *Indopodisma kingdoni* (Uv.); **32** *Squaroplatacris elegans* Zheng et Cao; **33** *Sinopodisma tsaii* (Chang); **34** *Fruhstorferiola omei* (Rehn et Rehn); **35** *Calliptamus barbarus* (Costa).

**Figures 36–42.**
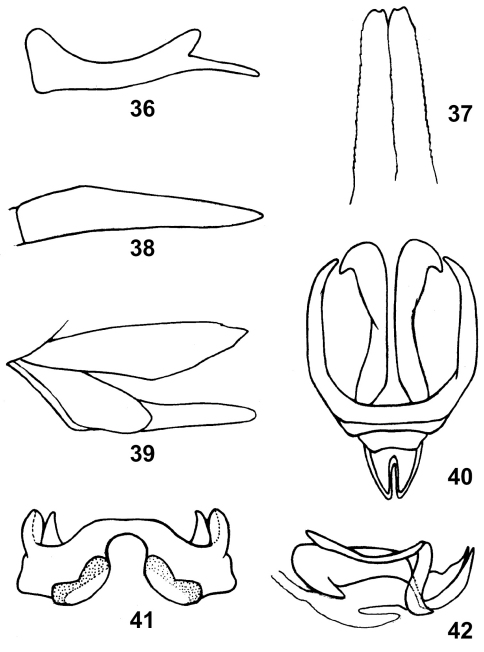
**36.** *Assamacris longicerca* (Huang), cercus, male, lateral view; **37** *Anapodisma miramae* Dov.–Zap., upper ovipositor valve of female, dorsal view; **38** *Leptacris vittata* (Fabr.), subgenital plate of male, lateral view; **39** *Longzhouacris hainanensis* Zheng et Liang, ovipositor, lateral view. **40–42** *Egnatius apicalis* Stål **40**: phallic organ of male, dorsal view **41** epiphallus dorsal view and **42** phallic organ, lateral view.

**Figures 43–47.**
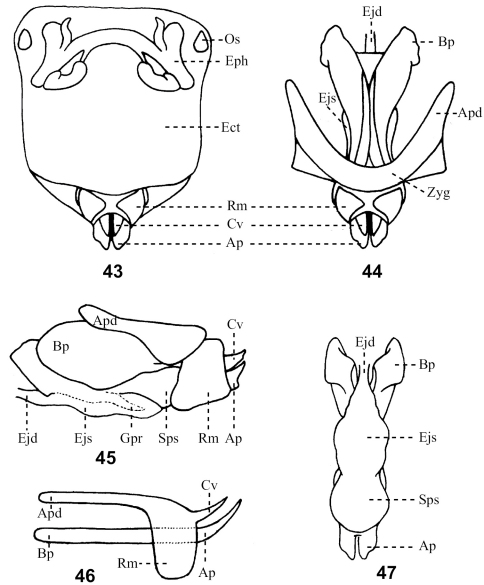
General morphology of phallic complex. Terminology and abbreviations used in the figures follow Dirsh (1956). **43** Whole phallic complex, dorsal view; **44** Phallic organ (phallic complex with epiphallus removed) dorsal view; 45. Phallic organ lateral view; **46** Penis and cingulum of simple form lateral view; **47** Phallic organ with zygoma apodems and rami removed ventral view. Abbreviations: **Ap** – apical valves of penis, **Apd** – apodemes, **Bp** – basal valves of penis, **Cv** – valves of cinglum and Rm – rami of cinglum **Zyg** – zygoma. Additional abbreviations for characters not coded: **Ect** – ectophalus **Ejd** – ejaculatory duct, **Ejs** – ejaculatory sac, **Eph** – epiphallus, **Gpr** – gonopore process, **Os** – oval sclerite of epihallus, **Sps** – spermatophore sac.

**Figures 48–53.**
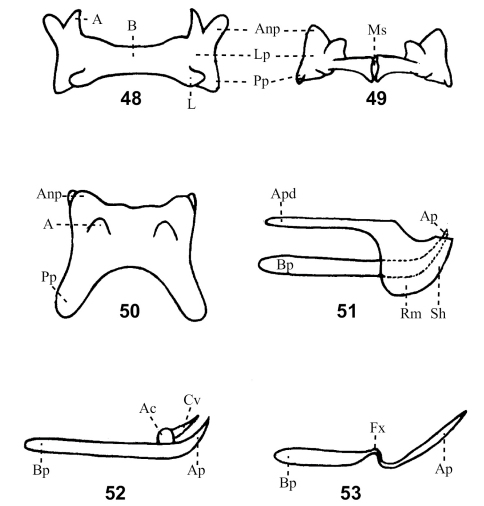
Variations of phallic complex: **48–50** Variation of epiphallus in dorsal view; 51. Penis with sheath formed from cingulum and exposed apex of penis; **52** Penis with arch of cingulum and valve derived from it; **53** Valve of penis with flexture. Abbreviations: **A** – ancora of epiphallus, **Ac** – arc of cinglum of phallic complex, **Anp** – anterior projections of epiphallus, **Ap** – apical valves of penis of phallic complex, **Apd** – apodeme of phallic complex, **B** – bridge of epiphallus, **Bp** – basal valves of penis of phallic complex, **Cv** – valves of cinglum of phallic complex, **Fx** – flexture **L** – lophi of epiphallus, **Ms** – median slit of epiphallus, **Pp** – posterior projections of epiphallus, **Rm** – rami of cinglum and **Sh** – sheath

## Appendix 1.

**Table:** **List of sampled taxa** († outgroups. *indicates genus not included in the final analysis. All ingroup genera are listed alphabatically).

**Genus**	**Species**	**Examined specimens**	
		♂	♀
*Egnatius* Voss.†			
	*apicalis* Stål	10	5
*Egnatioides* Liu†			
	*xinjiangensis* Liu	6	4
*Arcyptera* Serv. †*			
	*coreona* Shiraki	4	4
	*fusca fusca* (Pall.)	4	4
*Epacromius* Uv. †*			
	*tergestinus* (Charp.)		
*Alulacris* Zheng			
	*shilingensis* (Cheng)	11	8
*Anapodisma* Dov.-Zap.			
	*miramae* Dov.-Zap.	10	8
	*rufipenna* Zheng		2
*Anepipodisma* Huang			
	*punctata* Huang	1	1
*Apalacris* Walker			
	*hyaline* Walker	6	5
	*nigrogeniculata* Bi	5	5
	*tonkinensis* Ramme		1
	*varicornis* Walker	5	5
	*viridis* Huang et Xia	1	
	*xizangensis* Bi	14	11
*Armatacris* Yin			
	*xishanensis* Yin	1	5
*Assamacris* Uv.			
	*curticerca* ( Huang )	1	
	*longicerca* ( Huang )	6	12
*Bannacris* Zheng			
	*punctonotus* Zheng	2	2
*Calliptamus* Serv.			
	*abbreviatus* Ikonn.	15	10
	*barbarus* (Costa.)	15	10
	*coelesyriensis* (G.-T.)	7	2
	*italicus* (L.)	15	10
	*turranicus* Tarb.	7	15
*Carsula* Stal			
	*brachycerca* Huang et Xia		1
	*brachyptera* Huang et Xia	2	1
	*yunnana* Zheng		1
*Caryanda* Stal			
	*bambusa* Liu et Yin	3	3
	*bidentata* Zheng et Liang		1
	*elegans* Bol.	15	15
	*glauca* You	6	5
	*gracilis* Liu et Yin	2	10
	*hunana* Liu et Yin	2	3
	*methiola* Chang		1
	*nigrovittata* Lian et Zheng	4	3
	*omeiensis* Cheng		1
	*pieli* Chang	4	5
	*quadrata* Bi et Jin	1	1
	*vittata* Li et Jin	4	5
*Catantops* Schaum			
	*pinguis* (Stal)	10	7
	*simlae* Dirsh	2	2
*Chondracris* Uv.			
	*rosea brunneri* Uv.	6	8
	*rosea* (De Geer)	10	10
*Choroedocus* I. Bol.			
	*capensis* (Thunb.)	11	10
	*robusta* (Serv.)	13	10
	*violaceipes* Miller	15	10
*Conophyma* Zub.			
	*almasyi almasyi* Kuthy	10	10
	*zhaosuensis* Uv.	2	1
*Conophymacris* Will.			
	*chinensis* Chang	10	10
	*szechwanensis* Chang	10	10
	*viridis* Zheng	10	10
	*yunnanensis* Zheng	2	2
*Conophymopsis* Huang			
	*labrispinus* Huang	10	10
	*linguspinus* Huang	6	8
*Coptacra* Stal			
	*hainanensis*Tink.		1
	*tonkinensis*Will.	2	3
*Cuvipennis* Huang			
	*wixiensis* Huang	10	10
*Cyrtacanthacris* Walk			
	*tatarica* L.	10	7
*Dericorys* Serv.			
	*annulata roseipennis* (Redt.)	1	
	*tibialis* (Pall.)		
*Dimeacris* Niu et Zheng			
	*prasina* Niu et Zheng	2	2
*Ecphanthacris* Tink.			
	*mirabilis* Tink.	4	3
*Ecphymacris* Bi			
	*lofaoshana* (Tink.)	2	5
*Eirenephilus* Ikonn.			
	*longipennis* (Shir.)	10	7
*Epistaurus* I. Bol.			
	*aberrans* r.-W.	10	10
	*meridionalis* Bi	15	12
*Eucoptacra* I. Bol.			
	*binghami* Uv.	4	2
	*kwangtungensis* Tink.	10	11
	*motuoensis* Yin	5	6
	*praemorsa* Stal	5	5
*Eyprepocnemis* Fieb.			
	*hoktuensis* Shiraki	2	6
	*perbrevipennis* Bi et Xia		2
*Fer* I. Bol.			
	*bimaculatus* You et Li	4	4
	*nonmaculatus* Zheng		1
	y*unnensis* Huang et Xia	2	2
*Fruhstorferiola* Will.			
	*huangshanensis* Bi et Xia	6	11
	*huayinensis* Bi et Xia	3	3
	*kulinga* (Chang)	10	10
	*omei* (Rehn et Rehn)	1	5
	*tonkinensis* Will.	10	10
	*viridifemorata* (Caud.)	12	8
*Genimen* I.-Bol.			
	*burmanum* Ramme		1
	*yunnanensis* Zheng	7	4
*Gerenia* Stal			
	*intermedia* Br.-W.	1	1
*Gesonula* Uv.			
	*mundataszemaoensis* Cheng	3	3
	*punctifrons* Stal	8	6
*Habrocnemis* Uv.			
	*sinensis* Uv.	1	4
*Hieroglyphus* Krauss.			
	*annuliconis* (Shir.)	10	5
	*banian* (Fabr.)	13	7
	*concolor* (Walk.)		1
	*tonkinensis* I.-Bol.	10	3
*Indopodisma* Dov.-Zap.			
	*kingdoni* (Uv.)	7	10
*Kingdonella* Uv.			
	*hanburyi* Uv.	15	3
	*kozlovi* Mistsh.	14	13
	*nigrofemora* Yin	2	2
	*nigrotibia* Zheng		1
	*parvula* Yin	5	8
	*pienbaensis* zheng	1	1
	*qinghaiensis* Zheng		2
	*rivuna* Huang	3	1
*Lemba* Huang			
	*bituberculata* Yin et Liu	2	7
	*daguanensis* Huang	1	
	*viridatibia* Niu et Zheng	2	2
	*yunnana* Ma et Zheng	1	
	*zhengi* Li	2	
*Leptacris* Walk.			
	*taeniata* (Stal)	3	4
	*vittata* (Fabr.)	8	7
*Liaoacris* Zheng			
	*ochropteris* Zheng	2	4
*Longgenacris* You et Li			
	*maculacorina* You et Li	2	2
*Longzhouacris* You et Bi			
	*hainanensis* Zheng et Liang	4	5
	*jinxiuensis* Li et Jin	14	8
	*rufipenns* You et Bi	9	8
*Melanoplus* Stal			
	*frigidus* (Boh.)	4	7
*Miramella* Dov.-Zap.			
	*sinensis* Chang	2	1
	*solitaria* (Ikonn.)	5	3
*Niitakacris* Tinkham			
	*goganzanensis*Tink.	4	5
	*rosaeceanum* (Shir)	8	1
*Emeiacris* Zheng			
	*maculata* Zheng	2	2
*Ognevia* Ikonn.			
	*sergii* Ikonn.	2	1
*Oxya* Saerv.			
	*adentata* Will.	10	10
	*agavisa* Tsai	14	10
	*anagavisa* Bi	11	9
	*chinensis* (Thunb.)	12	10
	*hainanensis* Bi	11	10
	*intricata* (Stål)	10	10
	*ningpoensis* Chang	13	13
	*tinkhami* Uv.	13	12
	*velox*(Fabr.)	6	3
	*yunnana* Bi	8	10
*Oxyina* Hollis			
	*sinobidentata* (Hollis)	13	14
*Oxyrrhepes* Srtal			
	*cantonensis* Tink.	5	11
	*obtuse* (De Haan)		
	*quadripunctata* Will.		
*Oxytauchira* Ramme			
	*brachyptera* zheng	1	1
	*elegans* Zheng et Liang	2	
*Pachyacris* Uv.			
	*vinosa* (Walk.)	3	3
*Paratoacris* Li et Jin			
	*reticulipennis* Li et Jin	4	3
*Patanga* Uv.			
	*apicerca* Huang	1	1
	*humilis* Bi	12	10
	*japonica* (I.-Bol.)	10	7
	*succincta*(Johan.)	6	5
*Pedopodisma* Zheng			
	*emeiiensis* (Yin)	3	3
	*huangshana* Huang	1	1
	*protrucula* Zheng	4	4
	*shennongjiana* Huang	1	1
	*tsinlingensis* (Chang)	2	2
*Podisma* Berthold			
	*aberrans* Ikonn.	4	3
	*pedestris* (L.)	3	5
*Prumna* Motschulsky			
	*arctica* Zhang et Jin	10	12
	*cavicerca* Zhang	3	3
	*jingpohu* Huang	1	3
	*primnoa* F.-W.	10	10
	*primnoides* (Ikonn.)	3	
	*wuchangensis* Huang	1	1
*Promesosternus* Yin			
	*himalayicus* Yin	1	
	*vittatus* Yin		1
*Pseudoptygonotus* Zheng			
	*gunshensis* Zheng etal		1
	*kunmingensis* Cheng	7	6
*Pseudoxya* Yin et Liu			
	*diminuta* (Walk.)	15	15
*Pyramisternum* Huang			
	*herbaceum* Huang	1	1
*Qinlingacris* Yin et Chou			
	*elaeodes* Yin et Chou	3	4
	*taibaiensis* Yin et Chou	3	4
*Quilta* Stal			
	*oryzae* Uv.	7	8
*Shirakiacris* Dirsh			
	*brachyptera* Zheng	13	10
	*shiraki* (I.-Bol.)	15	8
	*yunkweiensis* (Chang)	9	6
*Sinacris* Tinkham			
	*longipennis* Liang	1	1
	*oreophilus* Tink.	1	1
*Sinopodisma* Chang			
	*bidenta* Liang	1	4
	*formosana* (Shir.)	5	4
	*houshana* Huang	2	2
	*huangshana* Huang		1
	*jiulianshana* Huang	2	2
	*kawakamii* (Shir.)	1	2
	*kelloggii* (Chang)	10	10
	*kodamae* (Shir.)	1	2
	*lofaoshana* (Tink.)	11	19
	*pieli* (Chang)	10	8
	*quizhouensis* Zheng	10	10
	*rostellcerca* Zheng et Liang	8	10
	*shiraki* (Tink.)	3	2
	*spinocerca* Zheng et Liang	1	2
	*splendida* (Tink.)	2	3
	*tsai* (Chang)	13	15
	*yingdensis* Liang	7	4
*Sinstauchira* Zheng			
	*gressitti* (Tink.)	1	1
	*pui* Liang et Zheng	11	11
	*ruficornis* Huang	10	10
	*yunnansis* Zheng	1	1
*Spathosternum* Krauss			
	*prasiniferum* (Walk.)	15	13
*Squaroplatacris* Liang et Zheng			
	*elegans* Zheng et Cao	4	3
	*violatibialis* Liang		1
*Stenocatantops* Dirsh			
	*splendens* (Thunb.)	15	10
*Stolzia* Will.			
	*hainanensis* (Tink.)	1	1
	*jianfengensis* Zheng et Liang	1	1
*Tauchira* Stal			
	*damingshana* Zheng	1	1
*Toacris* Tink.			
	*shaloshanensis*Tink.	1	1
	*yaoshanensis*Tink.	1	1
*Tonkinacris* Carl.			
	*decoratus* Carl.	1	1
	*meridionalis* Li	4	4
	*sinensis* Chang	10	8
*Traulia* Stal			
	*lofaoshana* Tink.	4	2
	*minuta* Huang et Xia	5	5
	*nigrotibialis* Bi	3	3
	*orientalis* Ramme	4	3
	*szetshuanensis* Ramme.	7	4
	*orchotibialis* Liang et Zheng	1	1
	*ornate* Shir.	4	4
	*tonknensis* C. Bol.	3	3
*Tristria* Stal			
	*palvinata* Uv.	1	1
	*pisciform* (Serv.)	1	
*Tylotropidius* Stal			
	sp.	2	3
	*yunnanensis* Zheng et Liang Ge-qiu	2	5
*Xenacanthippus* Mill.			
	*hainanensis* Tink.	4	1
*Xenocatantops* Dirsh			
	*brachycerus* (Will.)	10	8
	*humilis* (Serv.)	15	10
*Yunnanacris* Chang			
	*yunnaneus* (Ramme)	10	10
*Yupodisma* Zhang et Xia			
	*rufipennis* Zhang et Xia	2	2
*Zubovskia* Dov.-Zap.			
	*koeppeni* (Zub.)	4	3
	*parvula* (Ikonn.)	8	10
	*planicaudata* Zhang et Jin	5	3
	*striata* Huang	10	10

## Appendix 2.

**Character list**

1. Shape of body: (0) stout, ratio of body length to width equal at most 4; (1) moderate; ratio of body length to width is between 4–8; (2) elongated and cylindrical, ratio of body length to width is at least 8. (ordered)

**I. Head**

2. Obliquity of frons in profile: (0) not oblique, forming with vertex an right angle; (1) oblique, forming with vertex an acute angle of over 40º; (2) strongly oblique, forming with vertex an very acute angle less than 40º. (ordered)

3. Shape of fastigium in dorsal view: (0) normal, not strongly projected anteriorly, the distance from anterior margin of eyes to the apex of fastigium equal or less than the horizontal diameter of eye; (1) strongly projected anteriorly, the distance from anterior margin of eyes to the apex of fastigium obviously greater than the horizontal diameter of eye (Fig. 5).

4. Transverse groove at base of fastigium: (0) absent; (1) present and fine, not interrupting lateral carinae of vertex. (2) present and distinct, cutting through lateral carinae of vertex (Fig. 6). (ordered)

5. Interorbital distance of vertex: (0) obviously wider than the width of the frontal costa between antennae; (1) almost as broad as the frontal costa between antennae; (2) obviously narrower than the frontal costa between antennae. (ordered)

6. Foveola: (0) distinct; (1) absent or not perceptible.

7. Frontal costa between antennae: (0) not obviously projected; (1) obviously projected forward.

8. Shape of eye: (0) long oval, vertical diameter of eye greater than 1.3 times its horizontal diameter; (1) oval, vertical diameter of eye equal to or less than its horizontal diameter.

9. Size of eye: (0) large, vertical height greater than 1.3 times length of subocular groove; (1) small, vertical height less than 1.2 times length of subocular groove.

10. Shape of antennae: (0) filiform; (1) sward-shaped, width of basal segments greater than length.

11. Length of male antennae: (0) short, tip distinctly not reaching to base of hind femur; (1) long, tip distinctly reaching to or beyond base of hind femur.

**II. Mesosoma**

12. Convexity of median posterior margin of pronotum: (0) smoothly round or broadly angular; (1) projected into a right or acute angle.

13. Concavity of median posterior margin of pronotum: (0) not concave; (1) broadly concave (Fig. 12); (2) distinctly concave, forming a triangle (Fig. 11). (ordered)

14. Longitudinal margins of dorsal surface of pronotum: (0) constricted in the middle; (1) parallel.

15. Surface of pronotum: (0) smooth to finely sculptured; (1) coarsely granuate, irregularly carinulate, or tuberculate (Fig. 8).

16. Median carina on prozona of pronotum: (0) flat; (1) distinctly elevated and roundly pectinate (Fig. 9).

17. Distinctness of median carina on pronotum: (0) distinct, from almost complete to complete; (1) barely discernible to absent.

18. Median carina on pronotum in lateral view: (0) straight; (1) strongly elevated medially, forming a distinct round ridge (Fig. 10).

19. Incision on median carina of pronotum by principal sulcus: (0) shallow to indistinct; (1) very deep (Fig. 8).

20. Presence of additional incisions of median carina of pronotum by minor transverse carina(e): (0) absent; (1) present.

21. Ratio of length of prozona to length of metazona of pronotum measured along median carina: (0) 1.0–1.2; (1) 1.5–2.0; (2) more than 2.3. (ordered)

22. Lateral carinae on pronotum: (0) absent or slightly elevated, distinctly not reaching to posterior margin of pronotum; (1) distinctly elevated, complete or nearly so.

23. Ventral posterior angle of lateral lob of pronotum: (0) broadly round (Fig. 7); (1) roundly angular to anglular (Fig. 6, 10).

24. Posterior margin of lateral lob of pronotum: (0) not concave to slightly arched; (1) strongly concave.

25. Shape of prosternal process:  (0) conical (Fig. 16); (1) cylindrical (Fig. 17); (2) transverse and lobular (Fig. 20, 21); (3) mushroom-shaped (Fig. 18, 19).

26. (25:0) Apical part of cone-shaped prosternal process: (0) straight; (1) strongly bent posteriorly.

27. (25:1) Apical part of cylindrical prosternal process: 0 straight or slightly bent posteriorly; (1) strongly bent posteriorly, almost reaching anterior margin of mesosternum, (2) compressed laterally and flat apically.

28. (25:2) Ventral margin of lobular prosternal process: (0) truncate or slightly serrated (Fig. 21); (1) with 2–3 apically rounded, triangular processes (Fig. 20); (2) medially projected into a large triangle; (3) triangular as state 2 and turned anteriorly (Fig. 22, 23).

29. Anterior border of mesosternum: (0) straight or slightly arched; (1) broadly projected in the middle (Fig. 13).

30. Shape of mesosternal interspace: (0) wide, as long as or less than width; (1) elongate, length at least 1.3 times its narrowest width; (2) very reduced, lateral margins partly or completely contiguous. (ordered)

31. Contact of lateral lobes of metasternum medially: (0) separated; (1) contiguous.

32. Inner posterior corners of lateral lobes of mesosternum: (0) obtusely round or angularly round; (1) right angular to acutely angular.

33. Relative length of dorsal and ventral basal lobe of hind femur: (0) dorsal lobe as long as ventral lobe; (1) dorsal lobe longer than ventral lobe.

34. Shape of ventral genicular lobe of hind femur: (0) round or roundly angular distally; (1) spined distally.

35. Shape of dorsal genicular lobe of hind femur: (0) round distally; (1) spined distally.

36. Serration of dorsal carina of hind femur: (0) absent, smooth; (1) present, finely serrated.

37. Shape of distal end of dorsal carina of hind femur: (0) round or slightly broadly angular: (1) spined, acutely pointed, or narrowly obtuse-angular.

38. Outer apical spine on hind tibia: (0) absent; (1) present.

39. Number of spines on outer margin of hind tibia: (0) 5–6; (1) 8–10; (2) over 12. (ordered)

40. Distance between 1^st^ and 2^nd^ spines of inner spine series on hind tibia: (0) as long as any other inter-spine distance; (1) longer than any other inter-spine distance.

41. Distal half of hind tibia: (0) not obviously broaden toward apex, without obvious edges running through the spines; (1) broadened toward apex, with distinct outer and inner edges running through the spines; (2) strongly broadened toward apex, with sharp outer and inner edges running through the spines. (ordered)

42. Size of male tegmina: (0) developed, at least in contact with each other above abdomen; (1) abbreviated, lobate, and lateral, not in contact above abdomen, but reaching to posterior margin of metanotum; (2) rudimentary, not reaching to posterior margin of metanotum; (3) absent. (ordered)

43. Distal margin of tegmina: (0) round; (1) obliquely truncated.

44. Cells of distal part of tegmina: (0) rectangular or irregular; (1) oblique.

45. Radial cells in the middle of tegmina: (0) with irregular cross-veins; (1) with parallel cross-veins (Fig. 14).

**III. Metasoma**

46. Development of tympanal organ: (0) developed, distinct; (1) vestigial with just discernible opening, or absent.

47. Presence of tubercle on sides of apical field of male supra-anal plate: (0) absent, (1) present.

8. Presence of transverse groove on apical field of male supra-anal plate: (0) absent, (1) present.

49. Presence of transverse ridge on middle field of male supra-anal plate: (0) absent, (1) present (Fig. 25).

50. Presence of tubercle on sides of middle field of male supra-anal plate: (0) absent, (1) present.

51. Presence of transverse groove on middle field of male supra-anal plate in male: (0) absent, (1) present.

52. Presence of hair tufts on last sternum of abdomen: (0) absent; (1) present.

53. Presence and size of furcula: (0) absent; (1) present and small (Fig. 26); (2) present, and large and long (Fig. 28–30). (ordered)

54. Basal field of male supra-anal plate: (0) smooth without tubercles near lateral margins; (1) with two digitiform tubercles near lateral margins (Fig. 28).

55. Shape of male supra-anal plate: (0) triangular; (1) rectangular or trapezoid; (2) scutate.

56. Shape of male cerci: (0) conical; (1) compressed laterally.

57. (55:0) Length of conical cerci in male: (0) short; (1) long.

58. Curvature of male cerci: (0) straight; (1) curved inward posteriorly (Fig. 28); (2) curved upward posteriorly; (3) curved downward posteriorly.

59. Apex of cerci in males: (0) pointed; (1) round (Fig. 34); (2) truncated (Fig. 31); (3) bifurcated (Fig. 29); (4) dentate (Fig. 35). (ordered)

60. Shape of male cerci in lateral view: (0) strongly tapering toward apex, width at apical part less than at middle; (1) broadened toward apex, width at apical part slightly greater than at middle (Fig. 32, 33); (2) strongly broadened toward apex, width at apical part much greater than at middle (Fig. 35). (ordered)

61. Shape of male subgenital plate in ventral view: (0) very short, length equal to or less than basal width; (1) long, length greater than basal width, but not more than 1.5 times; (2) strongly elongated, more than twice basal width (Fig. 38). (ordered)

62. Shape of posterior part of male subgenital plate in ventral view: (0) conical; (1) trapezoid.

63. (61:0) Compression of posteriorly conical subgenital plate in males in ventral view: (0) not compressed; (1) compressed laterally.

64. Shape of male subgenital plate in dorsal view: (0) strongly tapering toward apex, end pointed or blunt; (1) gradually tapering toward apex, end round or concave; (2) not tapering, sometimes even slightly broaden, toward the apex, end truncated. (ordered)

65. Presence of tubercle at apex of male subgenital plate: (0) absent; (1) present and short (Fig. 29, 30); (2) present and much prolonged, forming prominent pointed projection. (ordered)

66. Posterior margin of female subgenital plate in ventral view: (0) triangularly projected posteriorly in the middle; (1) straight or broadly rounded.

67. Presence of lateral teeth on posterior margin of female subgenital plate in ventral view: (0) absent; (1) present.

68. Shape of dorsal valves of ovipositors in profile: (0) stout, less than 3 times as long as broad when in a position coalesced with ventral valves; (1) slender, more than 3.5 times as long as broad when in a position coalesced with ventral valves.

69. Serration of dorsal external margin of dorsal ovipositor valves: (0) smooth or weakly serrated; (1) distinctly serrated.

70. Presence of a notch on apex of dorsal external margin of dorsal valves of ovipositor: (0) absent; (1) distinctly present.

71. Apex of dorsal valves of ovipositor: (0) not bidentate; (1) bidentate.

**IV. Male genitalia**

Details of the male genitalia morphology are explained in Figures 43-53. Terminology for genital structures followDirsh (1956). Acronyms used in description of the listed genital characters are:

Ac: arc of cinglum (of phallic complex)

A: ancora (of epiphallus)

Ap: apical valves of penis (of phallic complex)

Anp: anterior projection (of epiphallus)

Apd: apodeme (phallic complex)

B: bridge (of epiphallus)

Bp: basal valves of penis (of phallic complex)

Cv: valves of cinglum (of phallic complex)

L: lophus (of epiphallus)

Rm: rami of cinglum (of phallic complex).

72. Rami of cinglum (Rm) of phallic complex: (0) undeveloped, narrowly sclerotized; (1) developed, broadly sclerotized (Fig. 45).

73. Length of the apodemes (Apd): (0) far from reaching to apex of the basal valves of penis (Bp); (1) reaching to apex of basal valves of penis; (2) reaching beyond apex of the basal valves of penis. (ordered)

74. Shape of apodemes (Apd) (dorsal view): (0) slender, more than 7 times as long as broad; (1) stout, less than 6 times as long as broad.

75. Prominence of arc of cingulum (Ac): (0) well developed and large; (1) weak, but perceptible; (2) absent. (ordered)

76. Bp and apical valves of penis (Ap): (0) connected by strongly scleorotized flexure (Fx) (Fig. 53); (1) separated, being connected by membrane.

77. Apex of Ap (in profile): (0) distinctly bent upward (Figs. 42); (1) straight; (2) distinctly bent sideward.

78. Length of the valves of cingulum (Cv): (0) very long, apex distinctly reaching beyond apex of Ap (Fig. 42) ; (1) long, apex reaching to or almost to apex of Ap; (2) reduced, apex reaching at most to middle of Ap; (3) completely absent. (ordered)

79. Shape of epiphallus: (0) bridge-shaped (Fig. 41); (1) shield-shaped (Fig. 49).

80. Integrity of epiphalus: (0) complete, not divided (Fig. 41); (1) longitudinally divided into two parts along midline, connected by membrane (Figs. 49).

81. Bridge of epiphalus in dorsal view in relation to width of lateral plate (width of plate refers to its width at ancora without including the latter): (0) broad; width in the middle broader than 1/2 of, but narrower than width of lateral plate (Fig. 49); (1) narrow; width in the middle narrower than half of the width of lateral plate (Fig. 41); (2) absent (Fig. 50). (ordered)

82. Presence of ancorae (A) and its size in relation to width of bridge of epiphalus: (0) developed, distinctly projected, longer than 1/2 of width of bridge (Fig. 41); (1) small, obviously less than 1/2 of width of bridge; (2) absent. (Ordered)

83. Development of lophi (L): (0) well developed, large; (1) undeveloped, small but perceptible; (2) absent. (ordered)

84. Shape of lophi: (0) lobiform with 2 or 3 lobes (Fig. 41); (1) lobiform with only one lobe.

85. Shape of anterior projections of epiphalus (Anp): (0) distinctly projected (Fig. 41); (1) slightly projected.

86. Posterior projections of epiphallus (Pp): (0) not or slightly projected; (1) distinctly projected.

87. Apex of ancorae: (0) pointed, (1) bluntly round; (2) truncated.

88. Length of Bp relative to Ap: (0) Bp more than 1.5 times length of Ap; (1) Bp as long as Ap (Fig. 42); (2) Bp less than 0.8 times length of Ap. (ordered).

## Appendix 3.

**Table:** Character Matrix (Taxa in bold are outgroups).

	CHARACTERS								
TAXA	1	11	21	31	41	51	61	71	81
***Egnatius***	1100000002	0000000001	0000----00	0000000010	0000000000	0000000000	0000000000	0010000100	10000000
***Egnatiuides***	1100000002	0000000001	0000----00	0000000010	0000000000	0000000000	0000000000	0010000100	10000000
*Alulacris*	0000110100	0101001000	-01000--11	0000001010	0100000000	101001-010	0000100010	1000001100	00000100
*Anapodisma*	0000110100	1011001000	200000--11	0000001010	0100001000	1021000000	0000100110	1000011100	11000002
*Anepipodisma*	0000000000	0011101000	200000--11	0000000000	03---10000	1010001000	0000000000	0111000100	01001100
*Apalacris*	0001-00000	---1000000	001000--11	0000011010	0010000000	10000010-0	0000000010	011-000001	00010100
*Armatacris*	0000110000	0100000000	001001--10	1100010010	0000001000	101101-100	2000000000	0101001100	11011101
*Aserratus*	0000010000	0001001000	101000--10	0000001010	03---00000	1010000100	0000000010	0020101100	00000100
*Assamacris*	0000011100	1101100000	101000--11	0000011010	0000000000	1010200031	0000000110	0111000000	00000000
*Bannacris*	0000210000	1101000000	101000--11	0000001010	0000000000	1010000200	0000100010	0001000100	10000000
*Calliptamus*	1100110000	010100000-	01001-0-11	0000011010	0000000000	100001-141	0000000000	011100-110	112--110
*Carsula*	2212000011	0101000000	10103---12	1000000120	1000100000	111001-000	1000000110	0111010101	020101-1
*Caryanda*	0001010000	---100-000	-01000---0	1001001110	1100000000	1110000000	00000--010	00-1000101	01000-1-
*Catantops*	0000210000	0101000000	00101-0-10	1000011010	0000000000	10-0000111	0000000000	00--000000	10000000
*Chondracris*	0000010000	0021100100	001001--10	0100010010	0000001000	100001-100	1000000000	0111000100	11010011
*Choroedocus*	0000010000	0101000000	1010111-10	0000011020	0000000000	101001-112	0000000000	0-01000000	-000110-
*Conophyma*	0000010000	0001001000	211000--11	0000000110	03---100--	-0-011-0-0	000-000000	0000211300	00010010
*Conophymacris*	0001010000	0101000000	111000--11	0000001110	0100000000	1000200311	00000-1010	0000000-00	00000000
*Conophymopsis*	0000010110	0001000000	20102--311	0000000110	03---10000	1020100000	0000000000	001021-300	10010110
*Coptacra*	0002100000	0021100000	001000--11	0000011010	0010000000	1020000100	0000000000	0110000101	00000100
*Curvipennis*	0000110000	0001101000	100000--11	0000001010	0100000000	1010000010	0000000010	0110001000	10000101
*Cyrtacanthacris*	0000110000	0100000000	0010111-10	0100010000	0000001000	1010000000	1000000000	0101000200	120101-1
*Dimeacris*	0002010000	1001000000	200000--11	1000000000	0100000000	1110101000	0000000000	0001010100	00010100
*Dericorys*	0100000000	0101110001	000000--10	0010010120	0000000010	0000001010	0000000001	0101011100	00010010
*Ecphanthacris*	0102000010	1021100110	001000--11	0000011010	0010000000	1010000000	0000000000	0100100100	00001100
*Ecphyacris*	0102100000	0021100000	001000--11	0000011010	0011000000	1010001000	00000--010	0110000101	00000100
*Eirenephilus*	0000010110	0101101000	000000--11	0000001010	0000000000	1010000000	0000100010	0011010100	00000100
*Emeiacris*	0000010000	0101001000	101000--11	0000000010	0000000001	0010001010	0000100010	0101011100	00010100
*Eokingdonella*	0000010110	0001001000	111000--11	0000000010	03---00001	0020001000	0000000010	0---------	--------
*Epistaurus*	0102200000	0101000001	001000--11	0000011010	0011000000	1020100-00	0000000000	0010001001	00000000
*Eucoptacra*	0102200000	0021000000	000000--11	0000011010	0011000000	1020000--0	0000000010	00-1000001	0000--00
*Eyprepocnemis*	0000010000	0101000000	-0101-0-10	0000011010	0000000000	1010000000	0000000000	011-000-00	10010100
*Fer*	0001010000	0101001000	10101-0-10	0001001110	1000100000	1110000000	0000000010	0100000301	01000110
*Fruhstorferiola*	0000110000	0101001000	001000--10	0000001010	0000000000	1010000012	00001-1010	01--00--00	00000100
*Genimen*	0000210100	1001001001	200000--11	1000000010	03---00000	10-0000000	0000000000	0110000300	00010100
*Gerenia*	1000000000	0021000000	001000--11	0000011010	0011000000	1010000000	0000000010	0100100200	11101100
*Gesonula*	0000-10000	0101001000	001001--11	1001001111	2000100000	11-0000-00	0000000010	01000-0101	00000100
*Habrocnemis*	1000000000	0101100000	10001-0-10	0000011010	0100000010	002001-000	0000000000	0101000101	20000000
*Hieroglyphus*	0001010000	1101001000	101000--10	1000001110	-000101000	11-0-001--	00000000-0	01010-1001	0000-000
*Indopodisma*	0000210000	0001001000	100000--11	0000001010	0200000000	102001-020	0000100010	0000111100	00000020
*Kingdonella*	1100010010	0--100-00-	110000--11	0000000010	03---10001	00-00010-0	0000000010	012--11100	11001100
*Lemba*	0001010000	0101000000	101000--10	0011001110	1000000000	1110000000	1001010010	0120100101	00000001
*Leptacris*	2202000001	0101000000	101000--12	1000000120	0000100000	1010001000	2010000000	0000011100	10100-02
*Liaoacris*	0000010100	0101001000	000000--11	0000000010	0000000000	1010001000	0000000010	0001011100	01000100
*Longgenacris*	0000110000	0101001000	101000--11	0000001010	0000000000	101001-010	0000100000	0000101100	10010101
*Longzhouacris*	0001110000	1101101000	10-000--01	1000011010	0100000000	1110100000	00000-1110	0001010100	10011101
*Melanoplus*	0000110100	0101001000	-00000--11	0000000010	0000000000	102001-010	0000100000	0100011100	00000100
*Miramella*	0000010110	1101001000	100000--11	0000000010	0-000000--	-020000000	0000200010	10-0101100	0100000-
*Niitakacris*	0000110000	0011001000	100000--11	0000001010	0100000000	1020000010	0000000010	0000111100	00000110
*Ognevia*	0000010110	0101000000	001000--11	0000000010	0000000000	1010000000	0000100010	0---------	--------
*Oxya*	0001-10000	0101001000	-01000--10	1001001110	20000000--	-1000000-0	00000--010	0100000-01	02000---
*Oxyna*	0001110000	0101001000	101000--10	1001000110	2000000000	11000000-0	0000000010	0---00--01	0000000-
*Oxyrrhepes*	2000000000	0101000000	001000--12	0000010120	0000000001	00100--100	1000000000	0---011100	00-10-01
*Oxytauchira*	0000010000	010100-000	10102--110	0001001110	1000000000	1110000000	0000010010	0100001101	00000100
*Pachyacris*	0000-00000	0101100100	001000--10	1100011010	0001001000	1000000100	1000000000	0111000100	11010011
*Paratoacris*	0001010000	0101001000	101000--10	1001001010	1000000000	1110000000	0000000010	0100000001	00000000
*Paratonkinacris*	0000010010	110100100-	101000--11	0000000010	0000000001	0010000310	0000100010	0---------	--------
*Patanga*	0000110000	0100000000	0010111-10	1100010010	0000001000	100001-0-0	1000000010	01--000100	01010011
*Pedopodisma*	0000110000	000100100-	-01000--10	000000-010	0200000000	1010000--0	0000100010	0100-1-100	01000100
*Podisma*	0000210110	0101000000	001000--1-	0000000010	0-00000000	1020201000	0000000010	0121110100	01000100
*Primnoa*	0000110110	-011001000	201000--11	000000-010	010000--00	1010---0-0	01-20000-0	0021014200	11010102
*Promeosternus*	0001010000	1101101000	101000--00	1000010000	010-000001	0020100111	0000000100	0000010100	00011110
*Pseudoxya*	0000210000	0101001000	101000--10	0011001110	2000000000	1110000000	0000000010	0100001101	01000010
*Pseudoptygonotus*	0000010000	0001000000	201000--10	0000000110	1100100000	1110200000	00000-1010	0100010101	00000011
*Pyramisternum*	0001110000	0101001000	10102--210	0001000110	1000000000	1100000000	0000000010	0---------	--------
*Quilta*	2000010000	0101001000	101101--00	0011101110	2000000010	0110000000	10010-1000	0111001001	020100-0
*Qinlingacris*	0000010110	0001000000	001000--11	0000000010	03---00000	1020001000	0000000010	002000-100	01000100
*Ranacris*	0000000000	0001100000	200000--11	0000011010	03---00000	1010000000	0000000---	----------	--------
*Shirakiacris*	0000010000	010100000-	01001-0-10	1000011010	0000000000	100001-111	00000-1000	0111001000	10010100
*Sinacris*	0000010000	0101001000	10102--110	0001001110	1000000000	1100000000	0000010010	0010001101	01001100
*Sinopodisma*	0000-10000	0001001000	101000--10	0000001010	0100000000	10100001--	0000100010	0000-1-100	00000-00
*Sinstauchira*	0002-10000	0101001000	10102--010	1000001110	1000100000	11-0000000	0000000010	0100000001	00001000
*Spathosternum*	0000010000	0101000001	01002--010	1000000110	0000100100	1010001000	0000000000	0110011000	01101121
*Squaroplatacris*	0000010000	0101000000	01101-0-10	1000001020	0000000000	101001-111	0000010000	0011000100	00001000
*Stenocatantops*	0000210000	0101000000	00001-0-10	1000011010	0000000000	1000000110	1000000000	0100000100	10000000
*Stolzia*	0000210000	-101101000	001000--11	0001001110	0000001000	1110000000	0000000010	0----0--01	00000-1-
*Tauchira*	0001010000	0101001000	10102--110	0001001010	1000100000	1110000000	0000000010	0100000101	01001100
*Toacris*	0002010000	0101001000	10101-0-10	1001001010	1000100000	1110000000	0000000010	0101000101	00000100
*Tonkinacris*	0000210000	1101001000	-01000--10	0000001010	0000000000	10100----0	0000100010	0100010100	00000100
*Traulia*	0000001000	-101000000	101000--1-	0000011010	0-00000000	10-00000--	00000000-0	01--000001	00000100
*Tristria*	2000010000	0101000000	11103---12	0000000120	0000000000	1020000100	1001000000	0101001100	00000000
*Traulitonkinacris*	0000011000	1101001000	101000--10	0000011010	1000000001	001021-031	0000000010	0---------	--------
*Tylotropidius*	0000010000	0101000000	01101-2-10	1000011020	0000000000	1010200000	0000000000	0011000100	10001001
*Xenacanthippus*	2212010001	0101001000	20101-0-12	1000000110	0000000010	0010000000	2010000010	0011210200	120100-2
*Xenocatantops*	0000210100	0100000000	00101-0-10	0000011010	0000000000	1010000010	0000000000	0110000000	100000-0
*Yupodisma*	0000010110	0001001000	101000--11	0000001010	0100000001	0021000000	0000100010	1001001100	00000001
*Yunnanacris*	0000210000	0001001000	100000--11	0000001010	0100000000	1010000010	0000100010	0000111100	01000101
*Zubovskia*	0000110000	0001001000	20-000--11	0000000010	03---1--00	10-0000010	0000-00010	1000111100	01000100
